# Phosphorylation Affects DNA-Binding of the Senescence-Regulating bZIP Transcription Factor GBF1

**DOI:** 10.3390/plants4030691

**Published:** 2015-09-16

**Authors:** Anja Smykowski, Stefan M. Fischer, Ulrike Zentgraf

**Affiliations:** ZMBP, General Genetics, University of Tuebingen, Auf der Morgenstelle 32, Tuebingen 72076, Germany; E-Mails: smyka@gmx.net (A.S.); stefan.fischer@zmbp.uni-tuebingen.de (S.M.F.)

**Keywords:** senescence regulation, GBF1, CASEIN KINASE II, phosphorylation, phosphorylation mimicry

## Abstract

Massive changes in the transcriptome of *Arabidopsis thaliana* during onset and progression of leaf senescence imply a central role for transcription factors. While many transcription factors are themselves up- or down-regulated during senescence, the bZIP transcription factor G-box-binding factor 1 (GBF1/bZIP41) is constitutively expressed in Arabidopsis leaf tissue but at the same time triggers the onset of leaf senescence, suggesting posttranscriptional mechanisms for senescence-specific GBF1 activation. Here we show that GBF1 is phosphorylated by the threonine/serine CASEIN KINASE II (CKII) *in vitro* and that CKII phosphorylation had a negative effect on GBF1 DNA-binding to G-boxes of two direct target genes, *CATALASE2* and *RBSCS1a*. Phosphorylation mimicry at three serine positions in the basic region of GBF1 also had a negative effect on DNA-binding. Kinase assays revealed that CKII phosphorylates at least one serine in the basic domain but has additional phosphorylation sites outside this domain. Two different *ckII* α *subunit1* and one α *subunit2* T-DNA insertion lines showed no visible senescence phenotype, but in all lines the expression of the senescence marker gene *SAG12* was remarkably diminished. A model is presented suggesting that senescence-specific GBF1 activation might be achieved by lowering the phosphorylation of GBF1 by CKII.

## 1. Introduction

Onset and progression of leaf senescence is accompanied by a massive change in the transcriptome, implying an important role of transcription factors in this process [1,2,3,4]. A temporal transcript profiling using microarrays with high resolution covering 22 time points of a defined single leaf of Arabidopsis during onset and progression of leaf senescence revealed a distinct chronology of processes. Promoter motif and transcription factor analyses revealed a differential activation of regulatory genes that influence gene expression at different time points during senescence progression [4]. Obviously, the two transcription factor families, WRKY and NAC factors, which largely expanded in the plant kingdom are both overrepresented in the senescence transcriptome of Arabidopsis and several members of both families have already been characterized as playing important roles in senescence regulation in Arabidopsis and other plant species [5,6,7,8,9,10,11,12,13,14]. Family members of both groups have been shown to react to elevated levels of reactive oxygen species (ROS), especially hydrogen peroxide at the transcriptional level, and are at the same time involved in regulating the intracellular concentrations of these molecules [7,8,9,10,11,14]. Remarkably, response to ROS was one of the earliest features identified in the high-resolution temporal chronology of processes [4].

Consistent with this observation, a tight regulation of the activity of the hydrogen peroxide scavenging enzymes catalase (CAT) and ascorbate peroxidase (APX) were detected in Arabidopsis, rapeseed and sunflower plants leading to elevated levels of hydrogen peroxide during bolting and flowering time, which is achieved on either the transcriptional or posttranscriptional level [15,16,17,18]. In Arabidopsis plants, transcriptional repression of *CAT2* by the bZIP transcription factor GBF1 at bolting time appears to be the initial step for elevation of hydrogen peroxide contents, whereas the loss of APX1 activity is not achieved on the transcriptional level. Inhibition of the APX activity is most likely mediated through its own substrate H_2_O_2_ in which APX is sensitive to its own substrate exactly in this developmental time window. APX regulation is therefore believed to build a positive feed-back loop to enhance the increase of hydrogen peroxide at this time point of development [15,18]. If transcriptional repression of *CAT2* is abolished in *gbf1* mutant plants, no elevation of the hydrogen peroxide concentration is seen, and consequently a delay in leaf senescence was observed [19]. However, expression analyses revealed that *GBF1* appears to be constitutively expressed in leaf tissue [19] suggesting posttranscriptional mechanisms for senescence-specific GBF1 regulation. GBF1 belongs to the bZIP family of transcription factors, which consists of at least 75 bZIP members in Arabidopsis divided into 10 subgroups based on sequence similarity of the basic region and the presence of additional conserved motifs. These bZIP transcription factors play crucial roles in almost every response pathway, including light and stress signaling, pathogen defense, seed maturation and flower development [20]. Plant bZIP factors preferentially interact with *cis*-elements containing an ACGT core sequence, which can be found in C- and G-boxes [21,22]. The G-boxes drive gene expression in response to various exogenous triggers like light, anaerobiosis and endogenous signals such as abscisic acid or methyl-jasmonate [23].

bZIP factor activity is altered by different mechanisms, one of which is phosphorylation. Phosphorylation can stabilize proteins and diminish degradation, as in the case of HY5, a G-box binding factor of the H group [24]. Most bZIPs hold putative phosphorylation sites not only in their DNA-binding domain but also in the nuclear localization site, or in the case of GBF1, also in the multifunctional mosaic region [25,26]. Furthermore, it is known that a few bZIP factors, for example GBF2, are translocated into the nucleus upon light treatment. This translocation might be due to phosphorylation triggered by various signals [27]. Concerning the DNA-binding of transcription factors, phosphorylation has been shown to stimulate [28,29], abolish [30,31] or simply not affect the interaction of DNA and protein [32].

GBF1 in particular has been shown to be phosphorylated by a partially purified serine/threonine kinase of broccoli with many characteristics of CASEIN KINASE II (CKII) and the phosphorylation by this CKII-like protein was exclusively mapped to serines and had an enhancing effect on DNA-binding *in vitro* [28]. However, phosphorylation mimicry often had a negative effect on DNA binding of bZIP factors [25,31]. Meshi and coworkers were also able to show that the basic region of HPB-1a of wheat, which contains the same amino acid sequence in the basic region as GBF1, is phosphorylated mainly through a calcium-dependent protein kinase and this negatively modulated DNA-binding. Other regions outside the basic region of HPB-1a were also phosphorylated by CKII [31]. 

CKII α subunits affect multiple developmental and stress-responsive pathways in Arabidopsis including light signaling, flowering time and the circadian clock [33,34,35]. In plants, both α and β subunits of CKII are often encoded by multiple genes. In *Arabidopsis thaliana*, each subunit is encoded by four genes. While three of these slightly different α subunits localize to the cytoplasm and/or the nucleus where they mainly phosphorylate transcription factors, one α subunit (α4) is targeted to the chloroplast [36] and phosphorylates a plastid sigma factor (AtSIG6) [37]. At least 37 direct targets of CKII have already been identified in plants (for overview see [35]) but besides the bZIP factor GBF1, no other target indicates the involvement of CKII in senescence regulation. However, CKII has been shown to be involved in SA mediated phosphorylation of TGA2, another bZIP transcription factor, which also resulted in significantly reduced DNA binding *in vitro* [38]. Moreover, CKII is also involved in ABA responses in maize and Arabidopsis [39], and both hormones have been shown to be involved in early senescence regulation [3,8,10]. To test whether CKII is involved in senescence-specific activation of GBF1, we performed kinase assays with wild type and mutated GBF1. Since CKII is highly conserved throughout eukaryotes, we used mammalian CKII which could phosphorylate GBF1 and its substrate calf casein *in vitro*. Mutation of all serines in the DNA-binding basic domain (position −11/−15/−19) led to a much weaker phosphorylation signal compared to wild type GBF1 indicating that at least one of these serines is targeted by CKII but also means that other CKII phosphorylation sites exist outside the basic domain. Plant CKIIα subunit addition as well as phosphorylation mimicry led to a reduced DNA-binding to G-boxes in *in vitro* binding assays to promoter fragments of two direct target genes, *CAT2* and *RBCS1a*. *ckII* α subunit1 and −2 single mutants were analyzed for their senescence phenotype but no visible phenotype could be observed; however, expression of the senescence marker gene *SAG12* was clearly delayed in all mutant lines. 

## 2. Results

### 2.1. GBF1 Is Phosphorylated by CKII

**Figure 1:**
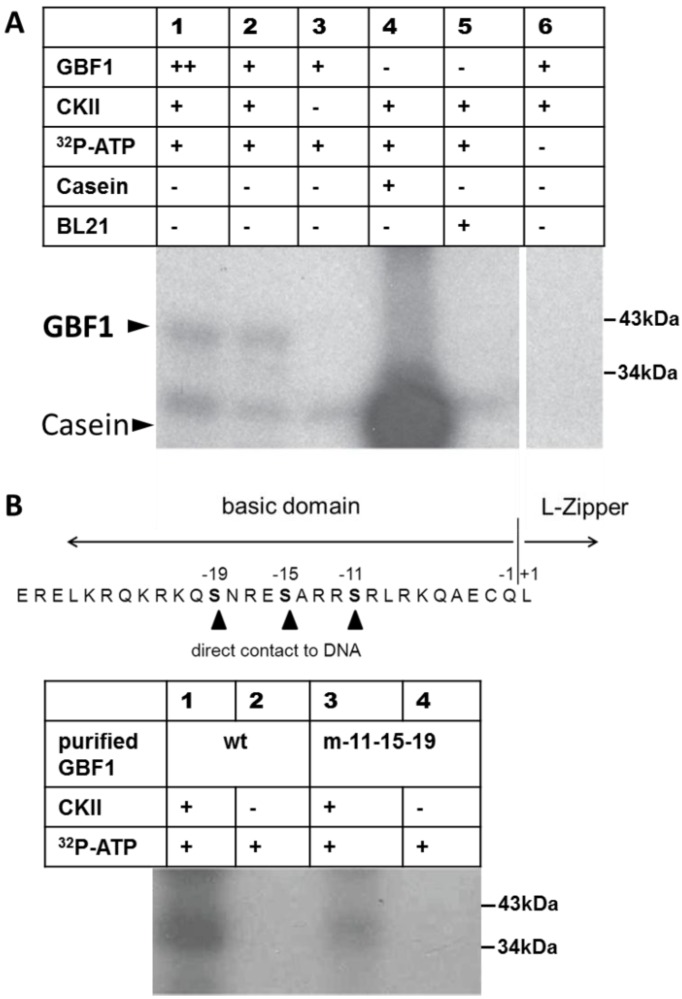
*In vitro* kinase assay with mammalian CKII. (**A**) *E. coli* BL21 raw lysate expressing recombinant GBF1 or no recombinant protein (BL21, lane 5) incubated with or without mammalian Casein Kinase II (NEB). Calf casein (Qiagen) was used as control substrate. Size in kDa is indicated on the right. ++: 50 μg, +: 25 μg raw protein lysate. All probes contained unlabeled ATP (100 μmol); (**B**) Purified 6×His-tagged GBF1 protein (wt) or mutated at position −11/−15/−19 of the basic domain (m−11−15−19) incubated with or without mammalian CKII (NEB).

Klimczak and coworkers were able to show phosphorylation of GBF1 by a CASEIN KINASE II-like protein complex isolated directly from broccoli [28]. CKIIs are highly conserved among eukaryotes and subunits can complement each other [40]. Maize subunits, for instance, have been shown to assemble with human CKII subunits to functional oligomeric forms [41]. Therefore, an *in vitro* kinase assay was conducted using a commercially available mammalian CKII (NEB). The mammalian CKII was able to phosphorylate recombinant GBF1 as well as its native substrate casein (Figure 1A). Phosphorylation of GBF1 was not detected without the addition of CKII or [γ-^32^P]-ATP. An additional band not corresponding to GBF1 is visible in all lanes, even where CKII is missing. This appears to be a protein phosphorylated by a bacterial kinase and can be designated as background signal of the raw BL21 bacterial lysate in this assay. The *in vitro* kinase assay was repeated using purified 6×His-tagged GBF1 protein (Figure 1B) and phosphorylation of mutated versions of GBF1 where all serines (position −11/−15/−19) in the basic domain were changed to aspartate. These three serines are highly conserved in nearly all plant bZIPs, and structural modelling revealed that these serines are in direct physical contact with the DNA [25]. The triple mutant version of GBF1 still showed a weak signal, demonstrating that CKII phosphorylates the basic domain as the signal is reduced but additional phosphorylation sites have to be present outside the basic domain as radioactivity is still incorporated.

### 2.2. GBF1 DNA-Binding Is Altered through Phosphorylation by CKII or Phosphorylation Mimicry

**Figure 2:**
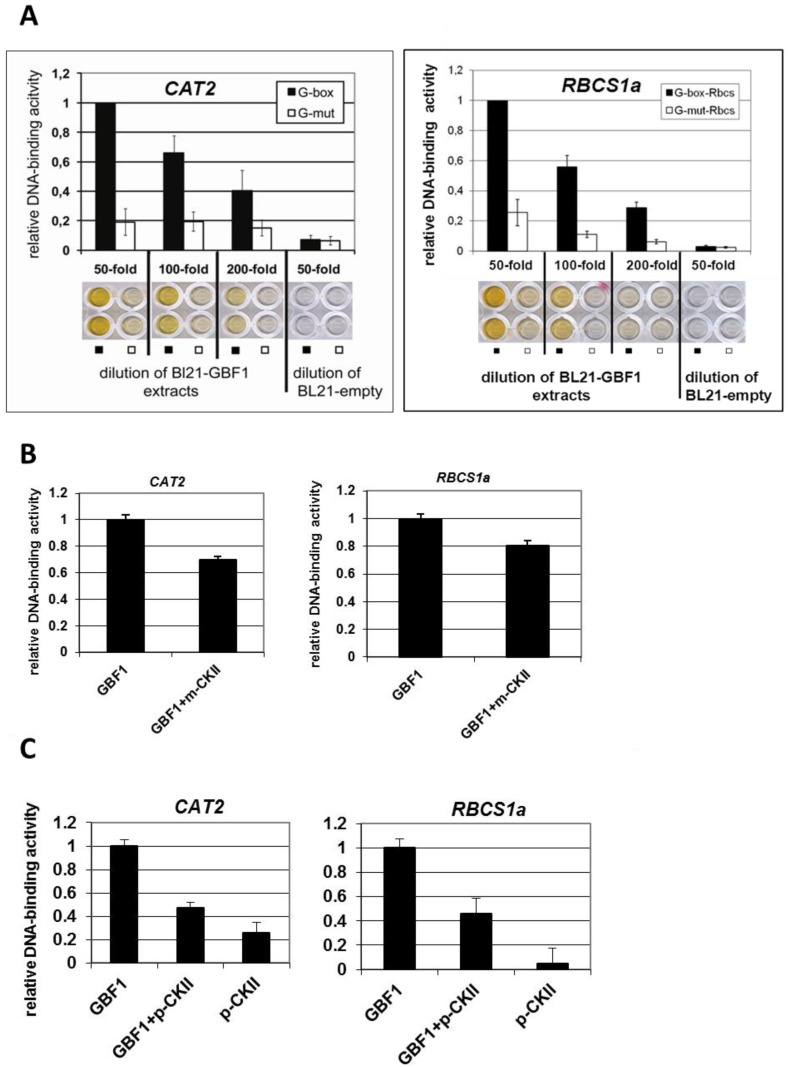
DPI-ELISA using recombinant GBF1 with the G-box of the *CAT2* and the *RBCS1a* promoter. Biotinylated plate coupled DNA fragments containing either the G-box (black bars) or a mutated version (G-mut; white bars) were incubated with crude *E. coli* BL21 extracts expressing recombinant 6×His-tagged GBF1 in different dilutions or the empty vector. Colorimetric reaction was quantified with a plate reader and plates were also photographed. The interactions between 50-fold diluted crude extracts and the G-box were set to 1.0, and all other interactions are expressed as relative values referring to GBF1/G-Box. (**A**) Concentration-dependent DNA-binding reaction of crude *E. coli* BL21 extracts expressing recombinant 6×His-tagged GBF1 with the G-box containing fragment of the *CAT2* promoter (left) or of the *RBCS1a* promoter (right); (**B**) DPI-ELISA after pre-incubation with or without mammalian CKII (m-CKII); (**C**) DPI-ELISA after pre-incubation with or without plant *CKII*α *subunit 2* expressed in *E. coli* (p-CKII). Error bars indicate standard deviation of 3–6 independent experiments.

DNA-binding assays were performed in the presence of CKII or with mutated versions of GBF1 in order to characterize the importance of phosphorylation of the GBF1 basic domain for its DNA-binding activity. An ELISA-based DNA-binding assay was established to analyze the DNA-binding of GBF1 to *CAT2* and *RBCS1a* promoter fragments, two direct target genes of GBF1 [19,42]. Biotinylated oligonucleotides containing either the G-box or mutated versions of the G-box of the *CAT2* promoter or of the *RBCS1a* promoter were attached to streptavidin coated ELISA wells, respectively. These plates were incubated with crude protein extracts of bacteria expressing recombinant 6×His-tagged GBF1 in different dilutions. DNA-bound GBF1 was detected using Anti-His-antibodies conjugated with horse radish peroxidase and the substrate 1,2-phenylenediamine hydrochloride. G-Boxes of both promoters were bound by recombinant GBF1 in a concentration dependent manner whereas binding to the mutated versions of the G-Box was very restricted (Figure 2A). If CKII was added prior to the binding assay either as purified mammalian CKII or as recombinant plant CKIIα2, DNA-binding was diminished, indicating that phosphorylation by CKII has a negative effect on DNA-binding while addition of plant CKII alone without GBF1 had no effect (Figure 2B,C).

**Figure 3:**
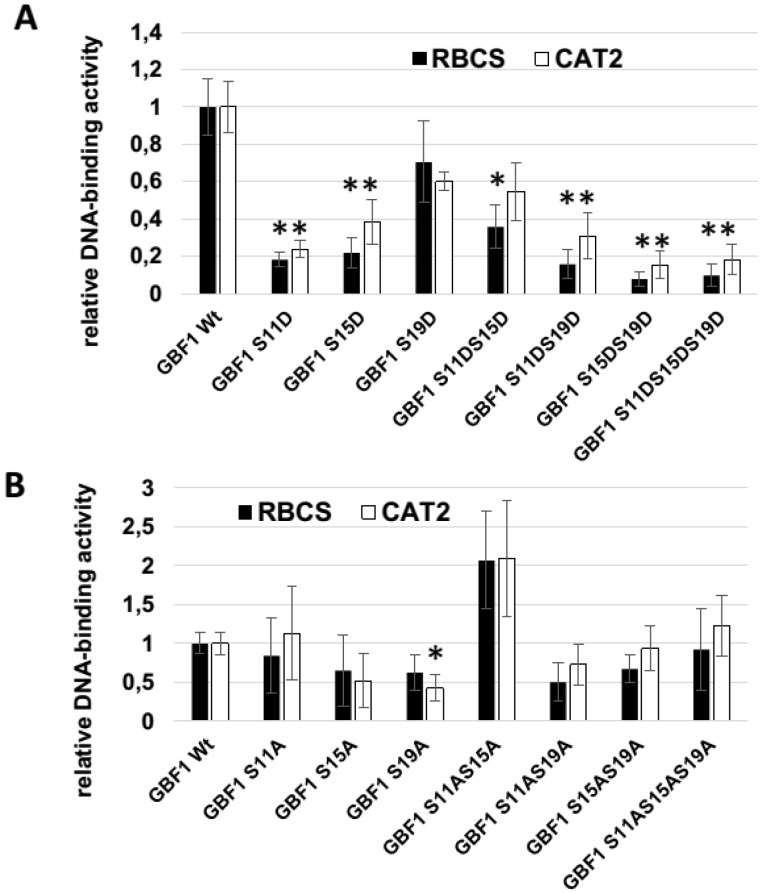
DPI-ELISA using recombinant GBF1 and single, double, and triple mutants of the serines in the basic domain with the G-boxes of the *CAT2* and the *RBCS1a* promoter. Biotinylated plate coupled DNA fragments containing the G-box of the *RBCS1a* promoter (black bars) or of the *CAT2* promoter (white bars) were incubated with crude *E. coli* BL21 extracts expressing recombinant 6×His-tagged GBF1 or GBF1 with substituted serines of the basic domain. The amounts of recombinant protein in the raw lysates were quantified on a Western blot using ImageQuantTL software, and DNA-binding was normalized to the protein amounts. The interactions between 50-fold diluted crude extracts and the wild type GBF1 was set to 1.0, all other interaction are expressed as relative values referring to GBF1/G-Box. (**A**) Consecutive substitution of serines at position −11, −15, and −19 with aspartate (D); (**B**) consecutive substitution of serines (S) at position −11, −15, and −19 with alanine (A). Error bars indicate standard deviation of 3–4 independent experiments, significant differences compared to wild type GBF1 are marked with asterisks (*t*-test, *p* < 0.001).

To investigate the role of the different phosphorylation sites in the DNA-binding domain, the three serines of this domain were mutated stepwise by site-directed mutagenesis to aspartate to mimic phosphorylation or to alanine to mimic the non-phosphorylated state. Successful mutagenesis was verified by sequencing the mutated plasmids. Single, double, and triple mutated versions were tested for their ability to bind to DNA. Raw extracts of *E. coli* BL21 cells expressing the different recombinant GBF1 versions were used in DPI-ELISAs with G-boxes of the *CAT2* and *RBSC1a* promoters, respectively. Expression of the 6×His-tagged recombinant proteins were analyzed by western blots (Figure S1), the amounts of recombinant protein in the raw lysates were quantified using ImageQuantTL software, and DNA-binding was normalized to the protein amounts (Figure 3). All exchanges to aspartate led to a significant decrease of the DNA-binding ability of GBF1 except of the S-19D exchange, which led to non-significant decrease of binding to both promoter fragments. If the same amino acids were changed to alanine significant changes were only observed in DNA-binding for the S-19A mutant version and the *CAT2* fragment. The S-11A/S-15A double mutant showed increased DNA-binding (not significant) but in this case the protein amount of the recombinant protein in the raw lysates was extremely high. Thus the increase in DNA-binding is most likely due to underestimated amounts of protein by the software leading to overestimated DNA-binding values. Taken together, the exchange of serine to aspartate has a clear inhibitory effect on DNA-binding, whereas the exchange to alanine has only moderate, non-significant effects. Since the exchange to aspartate introduces a negative charge to mimic phosphorylation, actual phosphorylation of the serines in the DNA-binding domain most likely leads to the inhibition of DNA-binding in which serine at position −11 is most and serine at position −19 least effective. The S-x-x-E/D-pS consensus binding motif of CKII was present only at position −15 but according to the analyses of CKII substrates of Meggio and Pinna [43] all three serines are in the range of possible targets for CKII phosphorylation (Figure S2). For the serine at position −19 also the exchange to alanine led to a significant decrease in DNA-binding, at least with the *CAT2* promoter fragment, possibly indicating that a mutation at this position in general leads to an inhibitory effect on DNA binding.

### 2.3. Senescence Phenotype of ckII*α* Mutant Lines

In order to analyze whether GBF1 phosphorylation via CKII has an effect on the progression of leaf senescence *in planta*, we characterized two different *ckII*α*1* and one *ckII*α*2* T-DNA insertion lines. T-DNA insertions were localized in exon or intron sequences in the *CKII*α*1* gene, respectively, and in an exon in the *CKII*α*2* gene. Plants which were homozygous for these insertions were identified by a PCR screen. No expression of *CKII*α*1* in *ckII*α*1*or *CKII*α*2* in *ckII*α*2* plants could be detected via RT-PCR.

**Figure 4:**
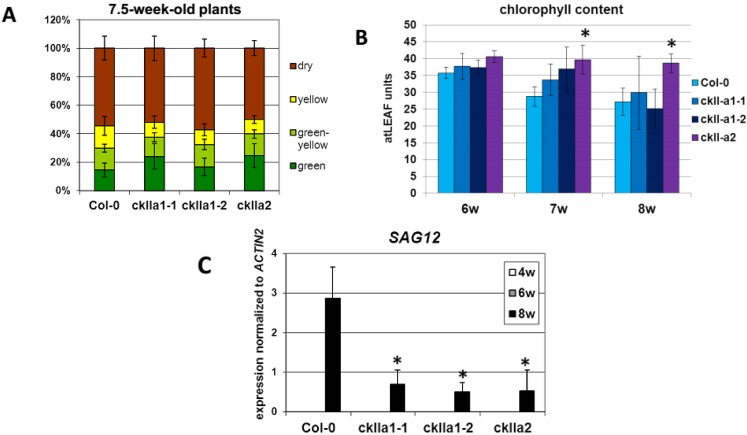
Senescence phenotypes of wild type Col-0, *ckII*α*1* and *ckII*α*2* mutant plants. Four plant lines were analyzed over development. (**A**) For a quantitative evaluation of leaf senescence, leaves of at least five plants were categorized in four categories according to their leaf color: (1) “green”; (2) leaves starting to get yellow from the tip as “yellow-green”; (3) completely yellow leaves as “yellow” and (4) dry and/or brown leaves as “dry”. The percentages of each group with respect to total leaf numbers are presented. (**B**) Chlorophyll content was measured using an *atLEAF+* chlorophyll meter over development in leaves No. 4 to7 of 6-, 7- and 8-week-old plants. Error bars indicate standard deviations of at least five plants. (**C**) Expression of the senescence marker gene *SAG12* encoding a cysteine protease was analyzed in leaf No. 6 and 7 of 4-. 6- and 8-week-old plant material by RT-PCR and normalized to the expression of *ACTIN2*. Error bars indicate standard deviations of four independent experiments, significant differences compared to wild type plants are marked with asterisks (*t*-test, *p* < 0.05).

These lines were grown side by side with wild type plants under long-day conditions for senescence phenotyping. Leaves of at least five plants were categorized into 4 groups according to their leaf color (fully green, green/yellow, fully yellow and brown/dry) (Figure 4A). A picture of a typical example of leaves of 7-week-old plants sorted with the help of a color code according to their age is presented in Figure S3. Furthermore, the chlorophyll contents of leaves No. 4 to 7 were measured by using an atLEAF+ chlorophyll meter (Figure 4B). In comparison to wild type plants, *ckII*α mutants showed no clear visible phenotype according to the sorted leaves and the percentage of leaves in the different categories. However, a delay in chlorophyll loss could be observed in *ckII*α*2* mutants, and expression of *SAG12* was clearly reduced in all mutant plants of the same age compared to WT (Figure 4C) suggesting a delay in senescence on the molecular level. *CAT2* and *RBCS1a* expression was also tested in 4- and 6-week-old plants, being the time point when these two target genes of GBF1 are downregulated in wild type plants and the transcription factor *WRKY53* is upregulated. Whereas *RBCS1a* and *WRKY53* expression was not significantly changed in both mutant lines compared to wild type plants, *CAT2* downregulation was impaired in the *ckII*α*2* mutant line, indicating that the different α subunit genes most likely have overlapping but also non-redundant functions (Figure S6). Moreover, intracellular hydrogen peroxide contents were measured in these plants lines over development in which the increase in H_2_O_2_ was slowed down in the *ckII*α*2* mutant line (Figure S7).

## 3. Discussion

Regulation of gene expression is the major regulatory mechanism underlying onset and progression of senescence implying a central role for transcription factors. Many of these are in turn regulated on the transcriptional level, but at some point specific factors have to be activated in a transcription-independent manner [1,2,3,4,5]. GBF1 appears to be one of these factors, since expression of *GBF1* is not altered in leaf tissue over development. GBF1 was shown to regulate the onset of leaf senescence most likely through the transcriptional down-regulation of the genes encoding the hydrogen peroxide scavenging enzyme *CAT2* and the small subunit of *RUBISCO* (*RBCS1a*). *gbf1* mutant plants maintained constant *CAT2* expression, thus lacking an increase in intracellular hydrogen peroxide and the induction of *SAGs* and consequently exhibited a delayed senescence phenotype [19].

One possible mode of posttranscriptional regulation is phosphorylation of transcription factors at specific sites leading to activation or inhibition of their activity. Klimczak and coworkers [28] partially purified a GBF1 phosphorylating activity of broccoli by chromatography on heparin-sepharose and DEAE-cellulose and characterized essential features of recombinant Arabidopsis CKII activity [28]. Reconstitution of Arabidopsis CKII from recombinant subunits to a tetrameric complex had properties very similar to those of the oligomeric CKII-like complex of broccoli, whereas the properties of the catalytic CKIIα1 subunit alone differed [44]. Since coexpression of the α and β subunits in *E. coli* lead to massive formation of inclusion bodies [44], we were not able to solubilize and reconstitute active tetrameric complexes. Since CKIIs are highly conserved among eukaryotes and subunits can complement each other [40], we verified GBF1 *in vitro* phosphorylation using mammalian CKII (Figure 1). Moreover, mutation of all serines in the DNA-binding basic domain (position −11/−15/−19, Figure 1B) led to a much weaker phosphorylation signal compared to wild type GBF1, indicating that CKII phosphorylation takes place in the DNA-binding domain but additional CKII phosphorylation sites appear to exist outside the basic domain, possibly in the proline-rich domain controlling gene transcriptional regulation or in the multifunctional mosaic region [25,26].

In order to see whether phosphorylation has an effect on GBF1 DNA-binding activity, we performed DNA-binding analyses in the presence of mammalian CKII and recombinant plant CKIIα2 subunits to both *CAT2* and *RBCS1a* promoter fragments (Figure 2). In animal cells, phosphorylation by CKII can inhibit the DNA-binding activity of transcription factor homodimers but not heterodimers [45]. Here, the addition of mammalian CKII as well as of a recombinant plant CKIIα2 subunit to GBF1 DNA-binding assays also inhibited DNA-binding. Phosphorylation mimicry by exchange of the serines to aspartate in the DNA-binding domain also revealed that DNA-binding of pseudo-phosphorylated GBF1 is always diminished but the degree depends on the site of phosphorylation and on the G-box it is binding to (Figure 3). The mutation of serine at position −11 seemed to be most effective as single mutant while −15 −19 double mutants and the triple mutant had the severest effect on DNA-binding to both *CAT2* and *RBCS1a* promoter fragments. Yet, serine at position −15 is the only serine which is surrounded by a motif identical to the CKII consensus binding sequence (Figure S2), but all three serines are possible CKII sites according to the CKII binding motif analyses of Meggio and Pinna [43]. Thus, GBF1 activity is most likely regulated through the interplay of different kinases which might also have different effects on DNA-binding. Exchange of the serines to alanine had no significant effect on the DNA binding except for serine at position −19 and the *CAT2* promoter fragment (Figure 3) suggesting that (i) the effect of exchanges to aspartate is most likely due to a phosphorylation mimicry and not a general effect of the mutation and (ii) the serine in position −19 appears to be more important for the overall structure of the GBF1 protein.

While Meshi and coworkers [31] claimed that phosphorylation of the basic region of the bZIP factor HBP1a of wheat which contains the same amino acid sequence in the basic region as GBF1 through calcium dependent kinases leads to a decrease in DNA-binding and Berberich and Cole could show that Casein kinase II inhibits the DNA-binding activity of Max homodimers [45], Klimczak and coworkers [28] clearly demonstrated that phosphorylation through CKII-like protein complexes isolated from broccoli increased DNA-binding of the Arabidopsis GBF1. Therefore, it is still unclear which effect phosphorylation of the basic domain has on bZIP function. Our results suggest that CKII phosphorylation using purified commercially available mammalian CKII protein as well as recombinant Arabidopsis CKIIα2 subunits has a negative effect on DNA-binding of GBF1 to the promoter fragments of two direct target genes of GBF1, *CAT2* and *RBCS1a* (Figure 2). Phosphorylation mimicry by exchange of the three serines at positions −11, −15, and −19 of the DNA-binding domain for which structural analyses proposed a direct contact to DNA [25] also led to a decrease in DNA-binding activity (Figure 3). This might be due to an inhibition of dimer formation of GBF1, which is a general prerequisite for bZIP activity. Even though phosphorylation mimicry was introduced only in the DNA-binding domain, DNA-binding and dimerization are two coupled processes influencing each other. Whether lower DNA-binding of phosphorylated monomers to DNA inhibits effective dimer formation or inhibited dimer formation between phosphorylated proteins inhibits DNA-binding still has to be elucidated. There is evidence for both models, dimerization can be observed either as protein-protein interaction without DNA [46] or dimer formation is initiated after sequential binding of the monomers to the DNA [47,48]. The monomer pathway allows faster assembly of certain bZIP dimer-DNA complexes and provides an efficient means of discriminating between specific and nonspecific DNA target sites [47]. 

CKII plays a critical role in various physiological processes such as light signaling, circadian rhythms, hormone responses, and flowering time. CKII is not regulated on the transcriptional level (Figure S5) but appears to be a housekeeping kinase which is also involved in very basic cellular processes like the cell cycle, DNA damage or translation, and is therefore also not regulated by a simple on/off switch but more in a dynamic way in which the activity of the catalytic CKIIα subunit is modulated by the regulatory β subunits [35].

In order to characterize whether CKII could be a candidate for the senescence-specific activation of GBF1, *ckII*α mutants were analyzed (Figure 4). In contrast to the well-defined tetrameric structure of animal and yeast CKIIs, plant CKII can also exists in a monomeric and an oligomeric form whose subunit composition is not well defined. The α-subunits are the catalytic subunits and are sufficient for the phosphorylation of GBF1 [44]. Therefore, T-DNA insertion lines for the two α-subunits, *CKII*α*1* (At5g67380) and *CKII*α*2* (At3g50000), which are mainly expressed in leaves [36] were used to characterize the role of CKII in senescence regulation. All three tested *ckII*α*1* and *ckII*α*2* knock-out lines showed no significant visible senescence phenotype, but chlorophyll loss was clearly delayed in the *ckII*α*2* mutant plants. Furthermore, the analyses of the expression of the senescence-associated marker gene *SAG12* resulted in a strong decrease in all *ckII*α knock-out lines compared to the Col-0 wild type line indicating a delay in senescence on the molecular level. This is in line with findings of Mulekar and coworkers [33] who also detected no clear visible phenotypes in single mutants. However, the double and triple mutants are delayed in flowering and are affected in diverse developmental and stress responsive pathways rendering senescence phenotyping extremely difficult or even impossible; therefore, these lines were not included in our analyses. Due to gene redundancy, expression analyses of the direct target genes of GBF1 revealed that in the single *ckII*α mutants not many differences are observed compared to wild type plants (Figure S6). No significant differences in the down-regulation of *RBCS1a* expression could be detected. Likewise, *CAT2* downregulation is not altered in *ckII*α*1* deficient plants but impaired in *ckII*α*2* mutants indicating that CKII might play a role in GBF1-mediated *CAT2* downregulation, that the different α subunits appear to be involved to different extends and that *CKII*α*2* deletion cannot be fully compensated (Figure S6). This is in agreement with the significant delay in chlorophyll loss and with a slower increase in hydrogen peroxide contents of the *ckII*α*2* deficient plants (Figure S7). However, these differences between the two direct target genes of GBF1 also indicate that GBF1 most likely operates not alone but in different higher order complexes on both promoters.

**Figure 5:**
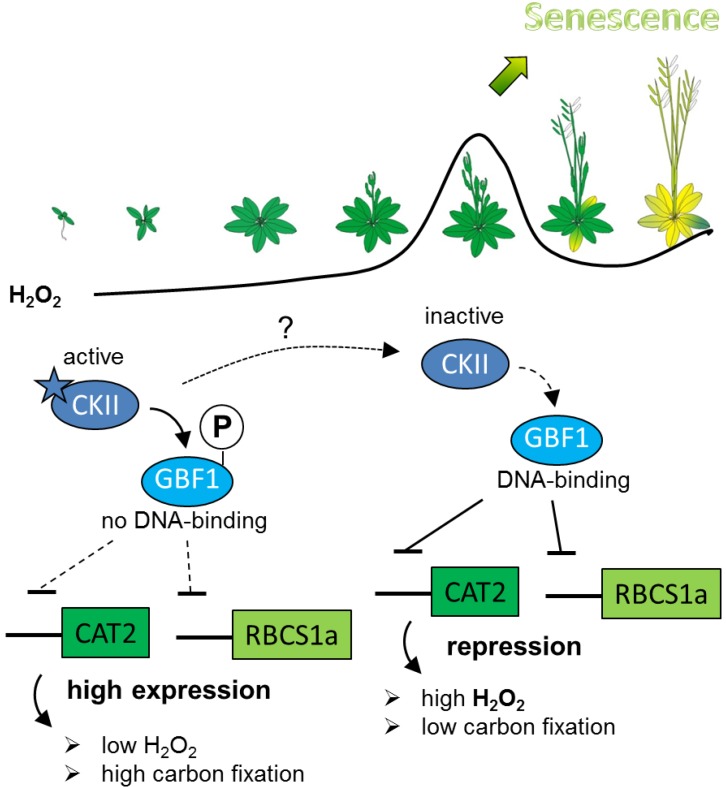
Model of senescence-specific activation of GBF1 by CKII phosphorylation postulating that CKII is active in young leaves and is inactivated during bolting time by a so far unknown trigger. Non-phosphorylated GBF1 can bind now to its target gene promoters *CAT2* and *RBCS1a* leading to the repression of these genes. Repression of *CAT2* and *RBSCS1a* then leads to an increase in intracellular hydrogen peroxide contents and low carbon fixation rates. Hydrogen peroxide was shown to be a signal to induce senescence.

Taken together, our results indicate that phosphorylation is affecting DNA-binding of GBF1 and that CKII might be one of the kinases involved in the senescence-specific regulation of GBF1. We propose the following model how CKII can be involved in GBF1-mediated senescence regulation (Figure 5). We postulate that in young leaves, the CKII kinase activity is high since CKII is also involved in very basic cellular processes like cell cycle, DNA damage or translation, and is therefore, as already mentioned, not regulated by a simple on/off switch but more in a dynamic way. By a so far unknown trigger, either activity or protein stability or translation efficiency of CKII decrease at bolting time, leading to a non-phosphorylated state of GBF1 which in turn can bind with higher affinity to the promoter of *CAT2* or *RBCS1a* leading to the repression of these genes. *CAT2* repression will lead to an increase in hydrogen peroxide content which is used as a signal to induce senescence, whereas *RBCS1a* repression will lead to a decrease in carbon fixation which is also observed already early in senescence. It is so far unclear which trigger could be involved in the inactivation of CKII but there are several links between CKII and different hormones like salicylic acid, abscisic acid or auxin [49,50,51]. In a complete *ckII*α knock-out line it could be expected that GBF1 would not be phosphorylated at the CKII specific sites and *CAT2* and *RBCS1* would be repressed all over development leading to high levels of hydrogen peroxide and low carbon fixation and thus very early senescence. However, this is not observed in the single mutants. If at all a delay of *SAG12* expression and for *ckII*α*2* deficient plants also a delay in chlorophyll breakdown and hydrogen peroxide increase can be observed. Due to gene redundancy, no effects or only weak ones can be observed in the single mutants, but it can be speculated that the inactivation of specific α subunits might be more efficient than those of others so that in the *ckII*α*2* mutants inactivation of CKIIα1 and 3 would be delayed leading also to a delay in senescence signaling. Moreover, high levels of hydrogen peroxide from early on can lead to compensatory effects like observed for *cat2* and *cat3* mutant plants. These plants neither exhibited increased levels of hydrogen peroxide nor showed accelerated senescence under normal long day conditions [52].

## 4. Experimental Section

### 4.1. Plant Material

*Arabidopsis thaliana* (L. Heynh), ecotype Columbia plants were grown in climatic chambers at 22 °C under long day conditions (16 h light/8 h dark) under moderate light intensity (60–80 μmol s-1 m-2). During growth and development of the rosettes, the leaves were color-coded with different colored threads. This allows that individual leaves could be identified according to their age even in very late stages of development. Leaves of the same positions within different rosettes were pooled. Seeds of T-DNA insertion lines in *CKII*α*1* (SALK_021073 in exon 4; SALK_131097 in intron 1) and *CKII*α*2* (SALK_126662 in exon 10) were obtained from the Nottingham Arabidopsis Stock Centre (NASC) and were characterized by PCR to be homozygous for the T-DNA insertion using gene specific and T-DNA left border primers (LBb1). No expression of *CKII*α*1* and *CKII*α*2* was detected in the knock-out lines by RT-PCR, respectively.

### 4.2. Senescence Phenotyping

For the evaluation of leaf senescence phenotypes leaves were sorted according to their age using a color code. In addition, leaves of at least five plants were categorized in four groups according to their leaf color: (1) “green”; (2) leaves starting to get yellow from the tip as “yellow-green”; (3) completely yellow leaves as “yellow” and (4) dry and/or brown leaves as “dry”. Chlorophyll content was estimated using an atLEAF+ chlorophyll meter. Each leaf was measured in triplicate and values were averaged. Leaves No. 4 to 7 of each rosette were measured.

### 4.3. Recombinant Proteins

The full length GBF1 cDNA fragment or the mutated versions were cloned into the pDEST42 expression vector (Invitrogen/Thermo Fisher Scientific, Waltham, MA, USA) to create pDEST42-GBF1 encoding the 6×His-tagged protein. *CKII*α*2* (AT3G50000) cDNA was also cloned into pDEST42 vector for expression. After cultivation of *E. coli* cells carrying the expression plasmids in LB broth and 1 mM IPTG treatment for induction of the recombinant protein syntheses, the cells were harvested, disrupted by sonication and centrifuged. The supernatants were used for experiments like Western blots, DPI-ELISAs and *in vitro* kinase assays. *In vitro* kinase assays were also conducted with purified proteins. Here, 500 mL of *E. coli* culture (BL21) expressing 6×His-tagged proteins was lysed in 2 mL LEW buffer (Macherey-Nagel, Düren, Germany), sonicated and centrifuged. The clear lysates were applied on Protino Ni-TED resin (Macherey-Nagel), washed with LEW buffer and eluted with elution buffer containing imidazol (Macherey-Nagel). The protein purification was verified by Western blot analyses followed by immune detection.

### 4.4. Western Blot Analysis and Immune Detection of 6×His-Tagged GBF1

10% SDS polyacrylamide gels were used for protein separation and were subsequently stained with *Coomassie* brilliant *blue* *R-250* *solution* (*BIO-RAD*) or transferred to nitrocellulose membranes using a semi-dry blotting procedure. After blocking for 1 h at room temperature in TBS-T (150 mM NaCl, 50 mM Tris, pH 7.6, 0.5% Tween-20) containing 5% (*w*/*v*) non-fat dry milk powder, the filters were gently shaken for 1 h in Anti-His antibodies solution (Qiagen, Hilden, Germany). Filters were washed three times in TBST for 10 min and then incubated with the secondary antibodies coupled with a horse radish peroxidase (Cell Signalling Technology/New England Biolabs GmbH, Leiden, The Netherlands). Again, the filters were washed 3 times in TBS-T for 10 min. Subsequently, antibody conjugates were detected by chemo luminescence. Quantification of the detected proteins was performed using the ImageQuantTL software (GE Healthcare Life Sciences, Cleveland, OH, USA).

### 4.5. DPI-ELISA

100 mL culture of *E. coli* cells (BL21) expressing 6×His-tagged GBF1 or mutated versions of GBF1 were centrifuged and lysed in 1 mL of lyses buffer (50 mM Tris, pH 7.5, 50 mM NaCl, 5 mM MgCl2, 1 mM PMSF, 0.02% NP40). Using this bacterial protein extract the DNA-binding assay was performed as described by Kirchler and coworkers [25]. Streptavidin coated ELISA wells (Nunc Immobilizer) were loaded with 2 pmol of the biotinylated *CAT2* promoter fragment containing the wild type (5′ ctcatca**cacgtg**gaatcc 3′) or mutated G-box (5′ ctcatca**aaaaaa**gaatcc 3′) or a *RBCS1a* promoter fragment also containing a G-Box (5′ aattatcttc**cacgtg**gcattattcc 3′) or a mutated version (5′ aattatcttc**ccagtg**gcattattcc 3′). These ELISA plates were blocked with 1% blocking agent (Roche, Mannheim, Germany). 2 μL of crude protein extracts (5 μg/μL) was diluted 1:50, 1:100, 1:200 in binding puffer and 60 μL were added to each well and incubated for 1 h. Subsequently, the raw lysate was removed and the wells were washed with blocking buffer (Qiagen) containing 0.1% Tween 20. After removing the washing buffer each well was incubated for 1 h with Anti-His antibody conjugates (Penta-His HRP, Qiagen) at room temperature. The OPD substrate (1,2-phenylenediamine hydrochlorid, Dako, Glostrup, Denmark) was added to the wells after intensive washing with blocking buffer (Qiagen), The detection reaction of the DNA-bound proteins was performed following the manufacturer’s protocol (Dako). Subsequently, the plates were read out at an absorbance of 492 nm using an ELISA plate reader (Tecan, Männedorf, Switzerland with filter setting at 650 nm for reference). Mammalian CKII and recombinant plant CKII were added to the 1:50 diluted protein extracts prior to DNA-binding and incubated for 30 min at 30 °C.

### 4.6. Site-Directed Mutagenesis

Site directed mutagenesis was performed following the protocol of the QuickChange II Site-Directed Mutagenesis Kit (Stratagene/Agilent Technologies, Santa Clara, CA, USA). As DNA template, the wild type version of *GBF1* was used. The primers were designed according to the manufacture’s advice (Table S1). Successful mutagenesis was verified by sequencing.

### 4.7. In Vitro Kinase Assay

Phosphorylation activities of mammalian Casein Kinase II (CKII, NEB) was assayed at 30 °C for 30 min in a final volume of 20 μL reaction buffer (10× Kinase Buffer, NEB) containing 200 μM ATP and 50 μCi [γ-^32^P]ATP (5000 Ci mmol^−1^) and one of the substrates with the protein amount of 25 or 50 μg. The kinase assay with purified GBF1 proteins was conducted using a final protein amount of 0.5 μg. Casein (25–35 kDa, Sigma-Aldrich, St. Louis, MO, USA) was used as a control protein. The reaction was terminated by the addition of 4× SDS sample loading buffer. After electrophoresis on 10% (*w*/*v*) SDS-PAGE, the phosphorylated substrates were visualized by autoradiography.

### 4.8. qRT PCR

RNA was isolated from pooled leaf material using leaf No. 6 and 7 with the PURESCRIPT RNA Isolation Kit (Gentra, Biozym). cDNA was polmerized using the iScript^™^ cDNA Synthesis Kit (Bio-Rad, Munich, Germany) according to the manufacturer’s instructions. For the qRT-PCR the iQ^™^ SYBR^®^ Green Supermix (Bio-Rad) was used following protocol of BIO-RAD. *ACTIN2* was chosen as reference gene for senescence since the variation of *ACTIN2* expression over different leaf and plant stages in Arabidopsis was very low in contrast to other housekeeping genes [53]. Expression of analyzed genes was normalized to *ACTIN2* expression according to [54]. The following primers were used (Table 1):

**Table 1:** Primers used for qRT-PCR.

	Forward Primer 5′–3′	Reverse Primer 5′–3′
GBF1	ggtcgaaagatggtgaagga	atccgattccaatcacgaag
CKIIa1	ttgatccacaactggaagca	cattaccatcatcatcatcatcag
CKIIa2	gcatttggtctcacctgagg	gaaaccggagggagtaataagaa
SAG12	cccggttaatgatgagcaagc	gctttcatggcaagaccaca
CAT2	caggttcgtcatgctgagaag	ttagatgcttggtctcacgtt
RBCS1a	attgcctacaagccaccaag	atttgtagccgcattgtcct
WRKY53	gatcacaagaacaccaccattagcc	aaagttgtgtcaatctcgaccgttg

### 4.9. Intracellular Hydrogen Peroxide Measurements

Hydrogen peroxide content was determined as described in Smykowski *et al.* [19]. Whole Arabidopsis leaves were detached and incubated for 45 min 10 μM 2′7′dichlorohydrofluorescein diacetate (DCFH-DA, Invitrogen/Thermo Fisher Scientific) at room temperature. Leaves were removed and rinsed in water, dried with filter paper and then frozen in liquid nitrogen. This material was either immediately ground or stored for a maximum of two weeks. Leaf tissue was homogenized in 1 mL of 40 mM Tris-HCI buffer pH 7.0, centrifuged at 20,600 g for 15 min and the supernatant was recovered. Fluorescence was determined using a spectrofluorometer (Berthold) using an excitation wavelength of 488 nm and an emission wavelength of 525 nm.

## 5. Conclusions

One of the major regulatory mechanisms governing plant senescence is transcriptional reprogramming governed by a high number of different transcription factors. Many of the senescence-regulating transcription factors are themselves induced or repressed on the transcriptional level; however, at some point, several transcription factors have to be regulated on the posttranscriptional level and GBF1 belongs to these factors. One possible regulatory mechanism is the activation or inactivation by phosphorylation by different kinases and CKII appears to be one of the senescence-regulating kinases modulating the DNA-binding activity of GBF1. But how the activity of CKII is controlled during development and onset of senescence and which other kinases are involved in the modulation of GBF1 activity still has to be elucidated. 

## Supplementary Files

Supplementary File 1
