# Correction: Phosphorylation Affects DNA-Binding of the Senescence-Regulating bZIP Transcription Factor GBF1. *Plants* 2015, *4*, 691–709

**DOI:** 10.3390/plants5030035

**Published:** 2016-08-31

**Authors:** Anja Smykowski, Stefan M. Fischer, Ulrike Zentgraf

**Affiliations:** ZMBP, General Genetics, University of Tuebingen, Auf der Morgenstelle 32, Tuebingen 72076, Germany; smyka@gmx.net (A.S.); stefan.fischer@zmbp.uni-tuebingen.de (S.M.F.)

The authors wish to make the following corrections to their paper [1].

1. In Section 4.3 (Recombinant Proteins) *CKII*α*2* (AT3G50000) cDNA was also cloned into pUC Spyne/Spyce for expression.
2. In Section 4.7. (In Vitro Kinase Assay), we used 5 and 15 µL of the protein extract containing the substrate equivalent to approx. 15 and 45 µg of total protein respectively, not 25 and 50 µg, and the reaction buffer contained only 100 µM ATP as indicated in the legend of Figure 1.
3. In Section 4.8. (qRT-PCR), unfortunately, the wrong method for the expression analyses was provided. We did not use qRT-PCR but semi-quantitative RT-PCR as indicated in the legend of Figure 4C. RNA was isolated from pooled leaf material with a PURESCRIPT RNA Isolation Kit (Gentra, Biozym) and cDNA was polymerized using the iScript™ cDNA Synthesis Kit (Bio-Rad, Munich, Germany) as indicated previously. Further, RT-PCR, rather than qRT, was performed using primer pairs as listed in Table 1. A low cycle number (25 cycles) was chosen so that the PCR did not reach saturation phase. Subsequently, RT-PCR products were separated on 1% agarose gels. The band intensity was quantified using the NIH image program (Scion Image, Scion Corporation). Expression was normalized to *ACTIN2* as previously indicated.
4. In Figure 2A error bars represent SE not SD, and in Figure 2C only two replicates were taken into account, so that the statement at the end of the figure legend that the error bars indicate SD of 3–6 independent replicates is not correct for all parts of the figure.

We apologize for any inconvenience caused to readers. The manuscript will be updated and the original will remain available on the article webpage.
