# Supplementary material for: Phosphorylation Affects DNA-Binding of the Senescence-Regulating bZIP Transcription Factor GBF1
Source: Plants (Basel). 2015 Sep 16;4(3):691–709. doi: 10.3390/plants4030691 (PMC4844403; doi:10.3390/plants4030691)
Supplement: Supplementary File 1 [file plants-04-00691-s001.pdf]

Supplementary Material

Table S1. List of primers used for site directed mutagenesis.

Name	Sequence 5'–3'	T_m [℃]
S17A F	aagactaccaaaccaacaGCTtcggctcaggaagttcct	81.6
S17A R	aggaacttcctgagccgaAGCtgttggtttggtagtctt	81.6
S17D F	aagactaccaaaccaacaGATtcggctcaggaagttcctccc	81.2
S17D R	gggaggaacttcctgagccgaATCtgttggtttggtagtctt	81.2
S120A F	aagaagtcaaaggggaacGCGaaaaaaaaggctgaagga	79.5
S120A R	tccttcagcctttttttCGCgttcccctttgacttctt	79.5
S120D F	ggcaagaagtcaaaggggaacGACaaaaaaaaggctgaaggaggt	79
S120D R	acctccttcagcctttttttGTCgttcccctttgacttcttgcc	79
S256A F	caacttcaacaaagagtagagGCTttgtcgaacgagaatcaaagc	79.4
S256A R	gctttgattctcgttcgacaaAGCctctactctttgttgaagttg	79.4
S256D F	caacttcaacaaagagtagagGATttgtcgaacgagaatcaaagc	78.5
S256D R	gctttgattctcgttcgacaaATCctctactctttgttgaagttg	78.5
S273A F	gagctacagagactctcaGCCgaatgtgataagctcaag	79
S273A R	cttgagcttatcacattcGGCtgagagtctctgtagctc	79
S273D F	gagctacagagactctcaGACgaatgtgataagctcaagtct	79.2
S273D R	agacttgagcttatcacattcGTCtgagagtctctgtagctc	79.2
S239A F	tccgctaggcggGCTagattgcggaagcag	81.6
S239A R	ctgcttccgcaatctAGCccgcctagcgga	81.6
S239D F	gaatccgctaggcggGATagattgcggaagcaggcc	82.2
S239D R	ggcctgcttccgcaatctATCccgcctagcggattc	82.2
S231A F	cagaagaggaaacaaGCTaaccgtgaatccgc	77.8
S231A R	gcggattcacggttAGCttgtttcctcttctg	77.8
S231D F	cagaagaggaaacaaGATaaccgtgaatccgc	73.4
S231D R	gcggattcacggtATCtgtttcctcttctg	73.4
S11S15A F	gaaGCCgctaggcggGCTagattgcggaagcag	81.1
S11S15A R	ctgcttccgcaatctAGCccgcctagcGGCttc	81.1
S11S15D F	gaaGACgctaggcggGATagattgcggaagcag	72.5
S11S15D R	ctgcttccgcaatctATCccgcctagcGTCttc	72.5

Positions that mutate the particular serines in the wild type are written in capital letters.

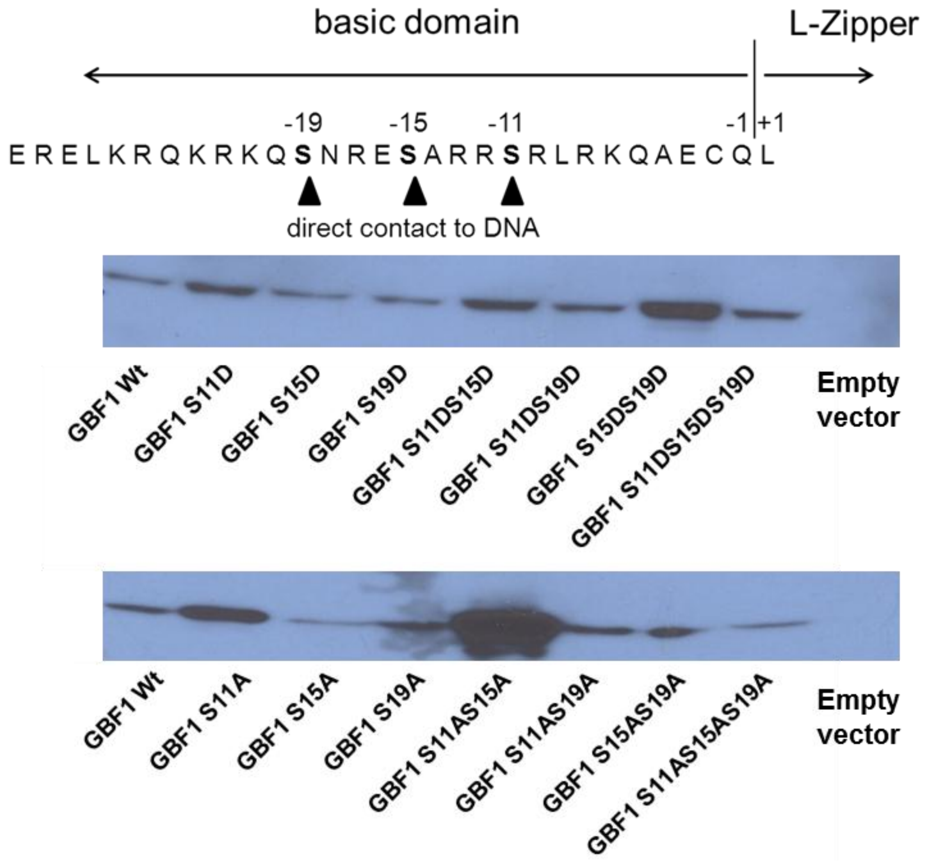

Figure S1. Western blot analyses with subsequent immune detection with anti-HIS antibodies of 25 µg of crude extracts of *E. coli* cells expressing recombinant 6xHis-tagged GBF1 wt or GBF1 in which the serines (S) of the basic domain were consecutively substituted with aspartate (D) or alanine (A), respectively. The empty vector was used as control.

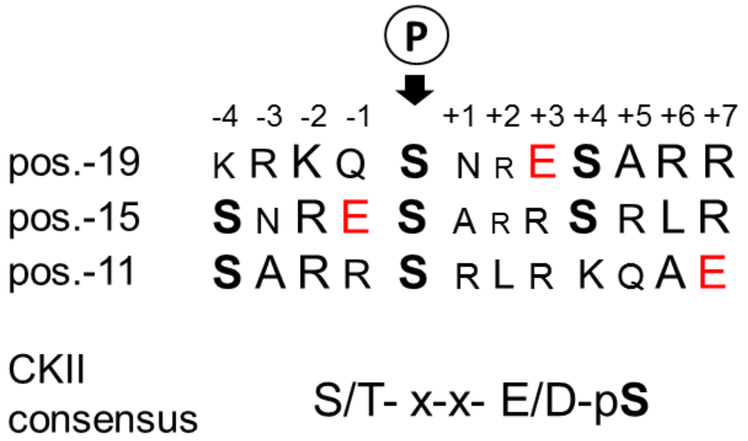

Figure S2. AA sequences surrounding the three serines of the basic DNA-Binding domain analyzed for their probability to be targeted by CKII. Serine at pos.-15 matches the CKII consensus binding motif, the size of the letters indicates the frequency of the amino acid to occur in CKII substrates according to Meggio and Pinna [43].

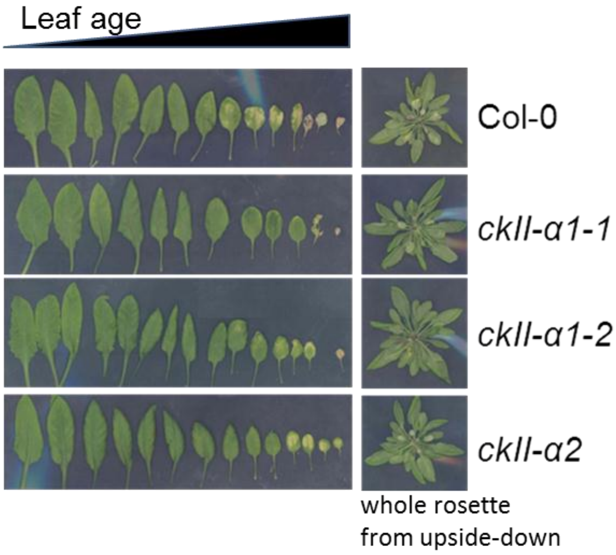

Figure S3. Leaves of 7.5-week-old wild type Col-0, *ckIIα1-1, ckIIα1-2* and *ckIIα2* mutant plant sorted according to their age. In order to visualize the older leaves of the rosette, whole rosettes were photographed from upside-down.

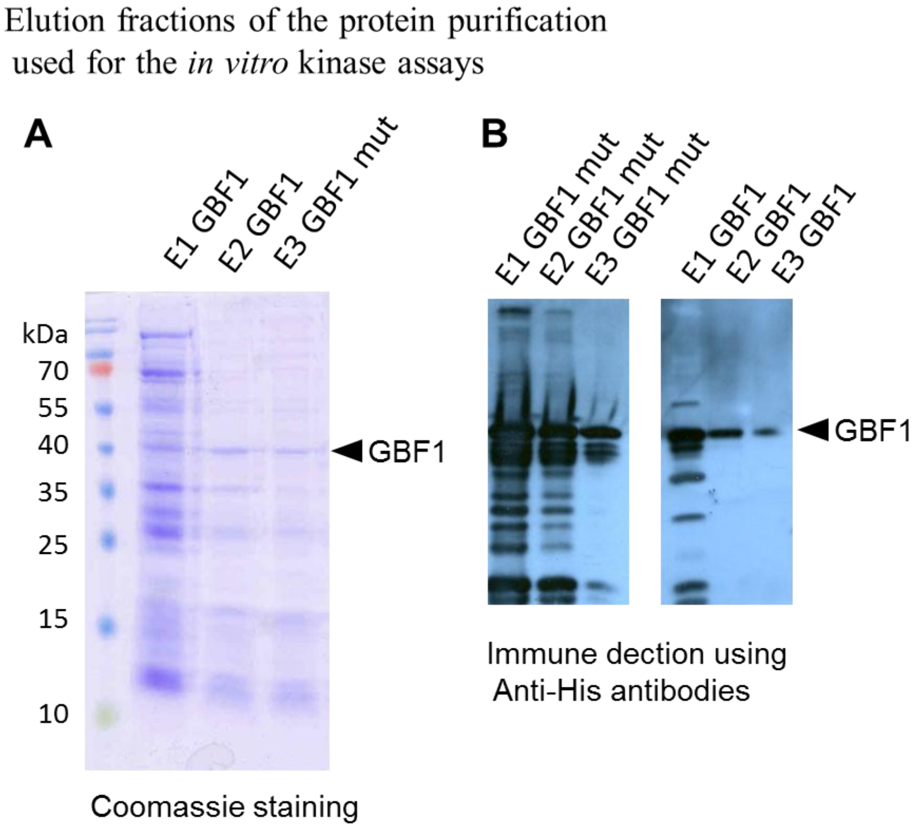

Figure S4. A Coomassie staining and **B** Western blot with subsequent immune detection using Anti-His antibodies of the elution fractions E1, E2 and E3 of the protein purification using 6xHis-tagged GFB1 and 6x His-tagged GBF1 3xD mutated version on Ni-TED Resin. Elution was performed using elution buffer containing imidazol (Macherey-Nagel, Düren, Germany). For the *in vitro* kinase assays, E3 of GBF1 mut (3xD) and E2 of GBF1 was used.

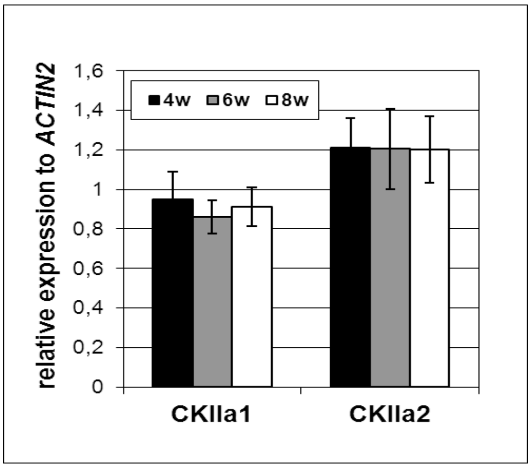

Figure S5. Expression of *CKIIα1* and *CKIIα2* was analyzed in Col-0 plants over development by RT-PCR and normalized to the expression of *ACTIN2*. Leaf No. 5 and 6 were pooled and analyzed. Error bars indicate standard deviations of four independent experiments.

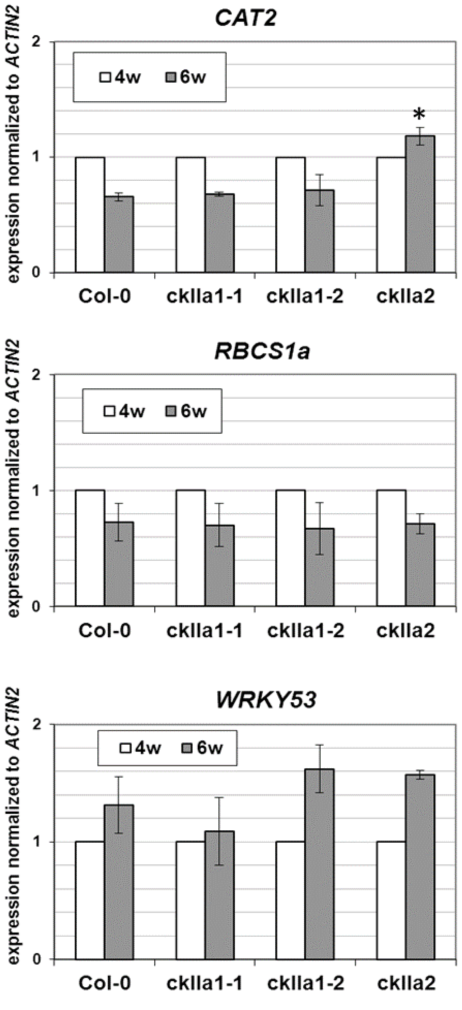

Figure S6. Expression of the two direct target genes of GBF1, *CAT2* and *RBCS1a*, as well as the senescence-related transcription factor *WRKY53* was analyzed by RT-PCR and normalized to the expression of *ACTIN2*. Leaf No. 5 and 6 were pooled and analyzed, the values of the 4 week-old plant material was set to 1 for each line to visualize the fold-change

in 6-week-old plant material for better comparison of the different lines. Error bars indicate standard deviations of two to four independent experiments, significant differences compared to wild type plants are marked with asterisks (t-test, $p < 0.05$).

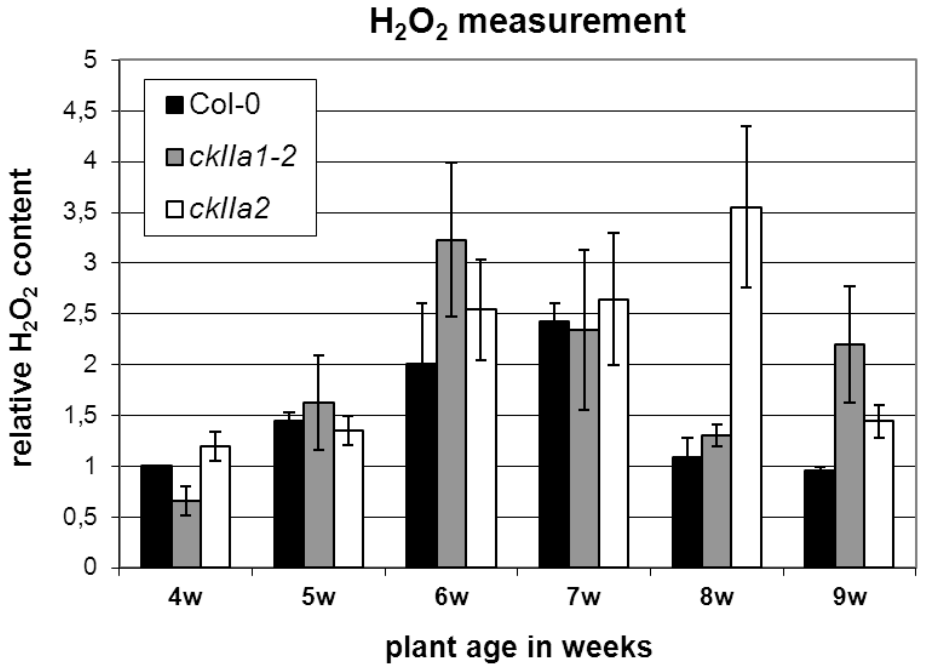

Figure S7. Intracellular hydrogen peroxide levels were measured using the fluorescent dye DCFDA in leaves of the different plant lines. Error bars indicate standard deviations of three to four independent experiments.
